# The critical events for motor-sensory temporal recalibration

**DOI:** 10.3389/fnhum.2012.00235

**Published:** 2012-08-08

**Authors:** Derek H. Arnold, Kathleen Nancarrow, Kielan Yarrow

**Affiliations:** ^1^Perception Laboratory, School of Psychology, The University of QueenslandSt. Lucia, QLD, Australia; ^2^Department of Psychology, City University LondonLondon, UK

**Keywords:** temporal recalibration, motor-sensory, adaptation, causality perception, time perception

## Abstract

Determining if we, or another agent, were responsible for a sensory event can require an accurate sense of timing. Our sense of appropriate timing relationships must, however, be malleable as there is a variable delay between the physical timing of an event and when sensory signals concerning that event are encoded in the brain. One dramatic demonstration of such malleability involves having people repeatedly press a button thereby causing a beep. If a delay is inserted between button presses and beeps, when it is subsequently taken away beeps can seem to *precede* the button presses that caused them. For this to occur it is important that people feel they were responsible for instigating the beeps. In terms of their timing, as yet it is not clear what combination of events is important for motor-sensory temporal recalibration. Here, by introducing ballistic reaches of short or longer extent before a button press, we varied the delay between the intention to act and the sensory consequence of that action. This manipulation failed to modulate recalibration magnitude. By contrast, introducing a similarly lengthened delay between button presses and consequent beeps eliminated recalibration. Thus it would seem that the critical timing relationship for motor-sensory temporal recalibration is between tactile signals relating to the completion of an action and the subsequent auditory percept.

It has been suggested that “time is an illusion. Lunchtime doubly so” (Adams, 1979; Chapter 2). Such a malleable approach to time perception might facilitate a bacchanal lifestyle, but it can also be adaptive, helping people cope with very real computational dilemmas when trying to determine if two sensory events are appropriately timed, such that they might be causally related.

The central nervous system must confront a number of problems when trying to determine event timing. For instance, some signals are subject to variable propagation speeds through the environment. The discrepant speeds of light and sound provide a good example. These dictate that visual signals concerning an event will reach our eyes *before* related auditory signals reach our ears. Moreover, the magnitude of this discrepancy is contingent on viewing distance (see Spence and Squire, 2003; Alais and Carlile, 2005; Arnold et al., 2005). Thus even before signals are encoded by the central nervous system, their physical arrival times represent only an ambiguous clue as to their precise temporal relationship.

The mismatch between physical timing and the times at which sensory signals reach our brain can be further exacerbated by characteristics intrinsic to the central nervous system. For instance, signals from your toes must travel further than signals from your fingers before reaching somatosenory cortex (Hirsch, 1862). Sensory signals can also propagate through the central nervous system at different and variable rates. One reason is that propagation speeds are generally related to signal intensity, thus a signal concerning a bright flash of light can propagate through the central nervous system more rapidly and reach cortex before a physically synchronous signal concerning a dim flash (Lennie, 1981; Burr and Corsale, 2001).

Despite assertions to the contrary (Dennett and Kinsbourne, 1992), the times at which sensory signals reach the brain are demonstrably important for time perception. A tap on your finger will typically seem to precede a physically synchronous tap on your toe (Von Békésy, 1963; Bergenheim et al., 1996) presumably because, as mentioned, signals from your fingers reach your brain *before* signals from your toes (Hirsch, 1862). Similarly, bright flashes of light can seem to precede physically synchronous dim flashes (Roufs, 1963; Wilson and Anstis, 1969), again presumably because of the relationship between signal intensity and neural propagation speeds. However, given the variability in times for sensory signals to reach cortex, sole reliance on activation times would provide a poor basis for determining timing in the external world. Instead, it is now well established that the brain also relies on malleable inferential processes.

If one is exposed to a stimulus containing systematically offset audio and visual signals for a protracted period, like a badly synched movie, the relative timing at which audio and visual signals seem synchronous can shift *toward* the adapted relationship (Fujisaki et al., 2004; Vroomen et al., 2004; Navarra et al., 2005; Vatakis et al., 2007; Hanson et al., 2008). Adapting to an audio lag, for example, can make visual signals and delayed sounds seem more synchronous than they did previously. This process is called temporal recalibration. Crucially, temporal recalibration might not reflect a universal recalibration of audio-visual timing perception. One can adapt to an audio lead of vision for one actor, and an audio lag of vision for another, resulting in simultaneous oppositely signed temporal recalibrations for the two actors (Roseboom and Arnold, 2011). This suggests that audio-visual temporal recalibration might help in daily life when interacting with multiple people at different distances.

While temporal recalibration was first shown for audio and visual signals, it has since been shown to influence all sorts of timing judgments, including all combinations of vision, audition, and touch (Hanson et al., 2008; Keetels and Vroomen, 2008), and even combinations of different types of visual event, which are obviously encoded within a single sensory modality (Arnold and Yarrow, 2011). Of most interest here, however, is an apparent change in our sense of causality evidenced by a recalibration between motor acts and contingent sensory events (Stetson et al., 2006).

The initial task used to explore motor-sensory temporal recalibration involved having participants repeatedly press a button that caused a beep. If a delay was inserted between button presses and the consequent beep, when that delay was taken away participants often felt as if the beep had happened *before* they instigated it by pressing a button (Stetson et al., 2006). Thus the sense that a person is responsible for a consequence (a beep) of their own actions (a button press) can be broken by having them adapt to an altered temporal relationship between action and consequence. Moreover, the magnitude of motor-sensory temporal recalibration was sharply reduced if, instead of intentionally pressing a button, beeps were repeatedly heard after buttons had been pressed against the participants' finger (Stetson et al., 2006). This suggests that intention is important for motor-sensory temporal recalibration, as evidently this effect is not simply driven by a systematic timing relationship between tactile perception and auditory input.

The discovery of motor-sensory temporal recalibration built on earlier observations, which had suggested that the apparent timing of the sensory consequences of an action might be brought into alignment with the instigation of the action (e.g., Yarrow et al., 2001, 2004a,b, 2006a,b; Yarrow and Rothwell, 2003). There is also a very similar illusion, called “intentional binding,” which is characterized by the apparent timing of an action and a consequent sensory event being drawn together (Haggard et al., 2002). However, unlike motor-sensory temporal recalibration (Stetson et al., 2006), intentional binding is assessed indirectly, with the timing of an action and its consequence judged relative to a rotating clock hand (Haggard et al., 2002). Importantly, a *reversed* effect can be obtained when unintended actions are evoked, by using transcranial magnetic stimulation to excite activity in motor cortex to induce an *involuntary* finger movement. In this case the apparent timing of involuntary actions and their sensory consequences were drawn apart (Haggard et al., 2002). As an intended causal relationship appears to be prerequisite for both intentional binding (Haggard et al., 2002) and for motor-sensory temporal recalibration (Stetson et al., 2006), the possibility exists that the timing of the neural processes responsible for volitional decisions is important for motor-sensory temporal recalibration, as opposed to the mere fact of a volitional decision.

Temporal contiguity offers a straightforward cue for inferring causality. Hence it is not surprising that motor-sensory temporal recalibration is critically dependent on the magnitude of the delay inserted between action and consequence. Haggard et al. (2002) found that intentional binding was much reduced for delays of ~650 ms, as opposed to delays of 250 ms (but see Humphreys and Buehner, 2009). Similarly, Stetson et al. (2006) found that motor-sensory temporal recalibration is maximal for delays of ~100 ms and is mitigated for increased delays, vanishing for delays of ~1000 ms. These data clearly show that there is a critical timing relationship for motor-sensory temporal recalibration, but what neural events are involved in this relationship?

To date, it has been established that intention is necessary for motor-sensory temporal recalibration and that the magnitude of motor-sensory temporal recalibration is critically dependent on the delay between an action and its perceptual consequence (Haggard et al., 2002; Stetson et al., 2006). Given these observations one might be tempted to conclude that the critical timing relationship for motor-sensory temporal recalibration is between the moment that one decides to act and the sensory consequence of that action. However, the implementation of an action involves a *sequence* of neural events. The decision to act itself is obviously related to a neural event(s), most likely in the basal ganglia and pre-supplementary motor cortex (Haggard, 2008). However, this decision initiates a train of pre-motor and motor cortex activity, and the timing of these processes could also be influential. Finally the sensation of having acted, e.g., the tactile sensation of having pressed a button, could also be critical.

To clarify the critical temporal relationship for motor-sensory temporal recalibration, we introduced a ballistic reach in-between the start of an action and its completion. By varying the extent of the reach we were able to manipulate the lag between decisions to act, the motor programming activity set in train by these decisions, and subsequent tactile sensations relating to action completion. We find that the precise timing of both decisions to act and of the motor planning required to act is inconsequential for motor-sensory temporal recalibration. Instead, the critical temporal relationship would seem to involve the termination of an action and the contingent auditory consequence of that action.

## General methods

A PC running Matlab (The MathWorks, USA) interfaced with a RX8 Multi I/O Processor (Tucker-Davis Technologies) was used to generate stimuli at a sample rate of 100 kHz. The RX8 Multi I/O Processor was used both to generate auditory stimuli and to record the timing of button presses. Audio stimuli were presented via Sennheiser HD595 headphones. Throughout each trial sequence participants listened to low volume white noise. This was done to mask any mechanical sounds made by button pressing during testing.

**Figure 1:**
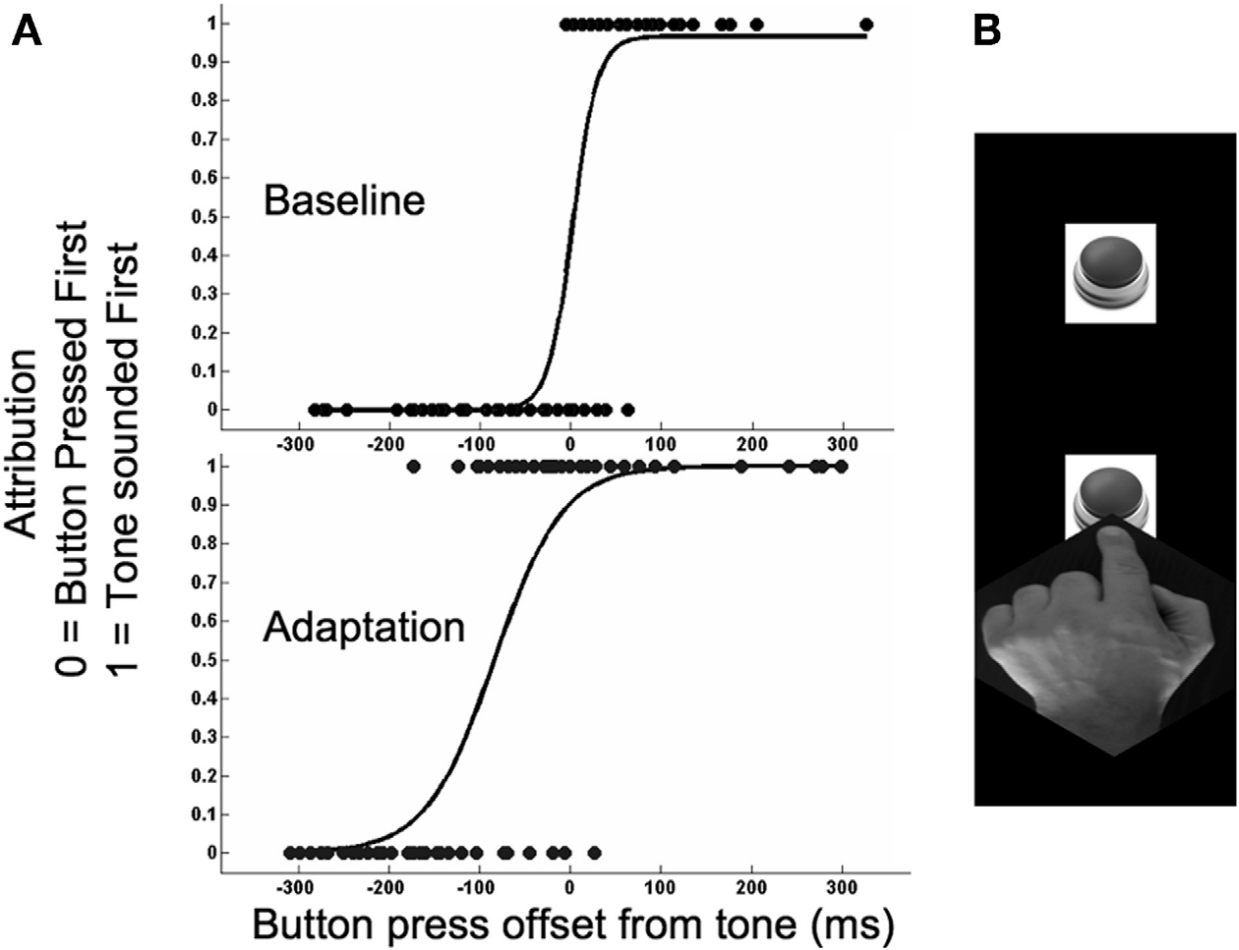
**(A)** Plots depicting when a participant felt they had pressed a button *before* they had heard a tone on the final sequence of a trial, expressed as a function of the physical timing differences between these events. Tones were presented at 0 ms. Negative values signify that the further button was pressed before the tone, whereas positive values signify that the further button was pressed after the tone. Data is depicted for a representative participant from baseline (above) and adaptation (below) runs of trials in Experiment 1a. **(B)** Depiction of experimental paradigm. This involved participants resting their finger on the nearer of two buttons and then, when they chose, reaching out as fast as possible to press the far button. During a trial this sequence was repeated up to 8 times. On all but the final sequence pressing the far button triggered a tone. On the final sequence the tone was not triggered by the participant pressing the far button but, after a delay, by the participant lifting their finger from the nearer button. This allowed us to sample tone presentation times that both preceded and lagged the final press of the further button. See Methods for further details.

Two buttons were positioned on a desk in front of the participant. Each experimental sequence began with participants resting their finger on the nearer of the two buttons and then, at a moment of their choosing, they reached out as quickly as possible to press the far button (see Figure 1). In different runs of trials the reach from the near to the far button was either 6 cm or 18 cm. A complete trial involved the participant repeating this sequence between 5 and 8 times, with the precise number determined at random on a trial-by-trial basis. Trials were self-paced, but participants were asked to leave at least 2 s between successive sequences and to rest their finger on the nearer button at the start of each sequence for at least 1 s. This was done to ensure that a trial represented a sequence of clearly segregated ballistic reaching movements.

On all but the last sequence of a trial pressing the far button caused a tonal pip (2 ms 400 Hz sine-wave tone) to sound at a time determined by the far button press. On these sequences the timing of tonal pip was predictable, determined by the times at which the participant pressed the far button. During baseline runs of trials this happened without delay whereas during adaptation runs of trials this happened after a systematic time lag. On each of these preliminary sequences the computer recorded the time taken from lifting the finger from the nearer button until it pressed the far button. We refer to this as “movement time.” On a final test sequence the tonal pip was triggered, after a delay, by the finger lifting from the nearer button. The timing of the tone on the test sequence was based on the average movement time during preliminary sequences on that trial (75, 100, or 125% of the average movement time). During a run of trials, test tone timing was manipulated according to the method of constant stimuli. Each of three sampled proportional test times were presented 20 times, for a total of 60 trials which were completed in random order. The physical timing of the far button press relative to the tone was recorded for each test sequence.

The completion of a trial was signaled by the white noise being silenced. Participants then indicated if they felt they had pressed the far button *before* the tonal pip during the test sequence (by pressing the near button, scored as 0) or after (by pressing the far button, scored as 1). As test tone times were based on preceding movement times, we were able to present tones both before and after far button presses. This differentiates our protocol from previous investigations of motor-sensory temporal recalibration (Haggard et al., 2002; Stetson et al., 2006). The duration of each reaching movement was variable, so a run of 60 trials could provide a distribution of 60 unique timing differences.

## Experiment 1

### Methods

In experiment 1 there were ten participants including the second author and an additional nine volunteers who were naive as to the experimental purpose. There were two reach conditions, with participants reaching either 6 cm or 18 cm to press the far button. Participants completed two runs of baseline trials and two runs of adaptation trials for each reach condition. On adaptation runs of trials, preliminary sequences had a 200 ms delay inserted between far button presses and tonal pip presentations.

Data from two runs of trials completed for each experimental condition were collated for each participant. Trials with timing differences greater than 300 ms were excluded from analysis, as these were usually caused by participants slipping and failing to press the further button at the end of a ballistic reach. For each participant this resulted in a single distribution of timing differences for each of the four experimental conditions. We fitted a logistic function to these and took the 50% point as an estimate of when the participant felt the further button press and tone had been synchronous on test sequences (see Figure 1). Individual estimates of recalibration magnitude were calculated for each reach condition by taking the difference between synchrony estimates from baseline and adaptation runs of trials.

### Results and discussion

**Figure 2:**
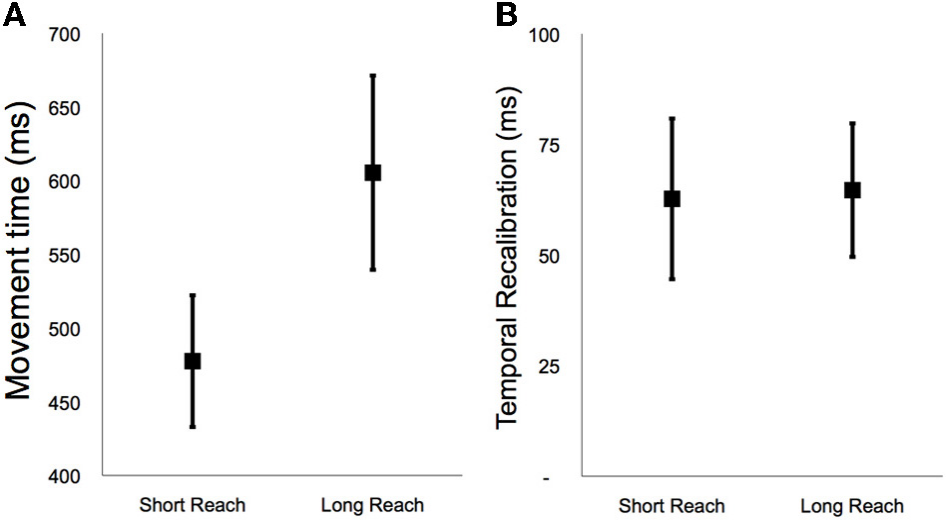
**(A)** Average movement time during short and long reach baseline conditions during Experiment 1. **(B)** Average temporal recalibration estimates during the short and long reach conditions of Experiment 1. Error bars depict ±1 SEM.

In Figure 2A we have depicted the average movement time from baseline trials for short and long reaches. These data show that far button presses were delayed by ~128 ms after a long as opposed to a short reach (paired *t*_9_ = 5.06, *p* < 0.0001). This means that in the long reach condition there was a greater temporal gap between decisions to act and the completion of that action (pressing the far button) than there was in the short reach condition. The long reach condition therefore also had a correspondingly greater gap between motor planning activity for ballistic reaching and action completion.

In Figure 2B we have depicted average recalibration estimates for short and long reach conditions. Note that recalibration was evident in both conditions, with participants thinking tones happened ~64 ms *earlier* relative to button presses after adaptation (Short reach 63 ± 18 ms, paired *t*_9_ = 3.85, *p* = 0.004; Long Reach 65 ± 20 ms, paired *t*_9_ = 4.28, *p* = 0.002). Thus adapting to a 200 ms lag for a tone after pressing a button made people think tones were happening *earlier* in relation to their button presses. Note, however, that there was no difference in the magnitudes of temporal recalibration for the Long and Short reach conditions (paired *t*_9_ = 0.13, *p* = 0.90). This shows that the extended delay for the Long reach condition, between deciding to press a button and actually pressing that button, had no discernable impact on recalibration.

While it is well established that motor-sensory temporal recalibration can break down if the delay between an action and its perceptual consequence is too great (see Heron et al., 2009, Figure 2; Stetson et al., 2006, Figure 4), it is equally well established that intent is critical for motor-sensory temporal recalibration (Haggard et al., 2002; Stetson et al., 2006). Thus the lack of variance in terms of temporal recalibration magnitude in Experiment 1 suggests two plausible interpretations. Either the additional 128 ms delay between initiating action and auditory feedback for longer reaching was too small to modulate motor-sensory temporal recalibration, or the moment that one initiates action is not critical for motor-sensory temporal recalibration. To tease these possibilities apart, in Experiment 2 we had people adapt to a 328 ms delay after a short reach, as opposed to the 200 ms delay in Experiment 1. This task therefore matched the long reach condition of Experiment 1 in terms of the delay between deciding to act and hearing a beep, but differed from both the long and short reach tasks of Experiment 1 in terms of the additional delay between completing an action (by pressing a button after a reach) and hearing a beep.

## Experiment 2

### Methods and results

Details for Experiment 2 were as for Experiment 1 with the following exceptions.

There were seven participants, including the second author and an additional six volunteers who were naive as to the purpose of the study. All participants had taken part in Experiment 1. Participants only made short reaches. During preliminary sequences in baseline runs of trials there was no delay between pressing the further button and tone presentations, but there was a 328 ms delay during adaptation runs of trials. In contrast to both the short and long reach conditions of Experiment 1, this adaptation protocol did not result in a reliable recalibration (23 ms ± 19 ms, paired *t*_6_ = 1.21, *p* = 0.27). As all participants had completed Experiment 1, we can contrast their average recalibration from Experiment 1 with their single recalibration estimate from Experiment 2. Doing so revealed that, for these participants, adapting to a 328 ms delay (Experiment 2), as opposed to a 200 ms delay (Experiment 1), resulted in a mitigated recalibration (Experiment 1, 66 ± 11 ms; Experiment 2, 23 ± 19 ms; paired *t*_6_ = 3.88, *p* = 0.008). These data refute the suggestion that an additional 128 ms delay, relative to the 200 ms delay in Experiment 1, would be insufficient to modulate recalibration magnitude. Rather, this additional delay was sufficient to largely eliminate recalibration.

## General discussion

Our data suggest that the critical neural events, in terms of their timing relationship, for defining whether motor-sensory temporal recalibration occurs are the sensation of having acted and the auditory consequence of that action. The separation of these neural events was unchanged by our major experimental manipulation, the length of a ballistic reach before completing an action. This manipulation did, however, further segregate decisions to act (and the prerequisite motor planning for acting) from the auditory consequence of acting. Thus the ineffectiveness of ballistic reach length strongly suggests that decision times and the timing of initial motor planning activity are inconsequential for motor-sensory temporal recalibration.

The ineffectiveness of ballistic reach length is informative because the additional delay it introduced between two events was demonstrably effective when inserted between two other comparable events. In Experiment 1 long ballistic reaches took ~128 ms longer than short reaches. During preliminary adaptation sequences in Experiment 2 we therefore extended the delay between button presses and hearing a tone by 128–328 ms from the 200 ms lag in Experiment 1. Thus, we extended the delay between completing an action and the auditory consequence of that action. This strongly modulated motor-sensory temporal recalibration, and thus suggests that the temporal relationship between *completing* a causal action and the perceptual consequence of that action is critical for motor-sensory temporal recalibration.

Previously it has been suggested that intent is crucial for motor-sensory temporal recalibration. If instead of pressing a button, the button effectively presses you, recalibration magnitude is much reduced (Stetson et al., 2006). Similarly, if a tone is caused by a voluntary action the time of the tone and the time of the action can seem to be drawn toward one another. Moreover, the opposite holds if a tone follows soon after an involuntary action (intentional binding; Haggard et al., 2002). Note, however, that these observations do not dictate that the *timing* of intention is critical. Rather, they may merely establish that intention, no matter when it is formed, is important for motor-sensory temporal recalibration and intentional binding. These observations are thus consistent with the implication of our data, that the critical neural events for motor-sensory temporal recalibration, in terms of their timing relationship, are the sensation of having finished acting and the perceptual consequence of having acted. Given the similarities between motor-sensory temporal recalibration and intentional binding, we suggest that our results are likely to apply in both contexts, although we have not demonstrated that here.

The ballistic action we used in this study (lifting a finger from one button and reaching to press a more distant button) can be described as a form of prepared interception. When triggered by an external event, it has been shown that these movements are governed by a motor programme prepared in advance of motion onset which is triggered ~150 ms prior to motion onset (see Marinovic et al., 2009). Here we would assume that the triggering event is information regarding a decision to act reaching criterion. Given that extending the time between motor pre-programming, motion onset and the sensory consequences of action had no impact on recalibration (see Experiment 1), we have concluded that the timing of the neural processes responsible for preparatory motor planning and decisions to act, were inconsequential for motor-sensory temporal recalibration in our study. Interceptive actions, however, are subject to on-line error corrections (Brenner et al., 1998; Tresilian and Plooy, 2006) and the timing of neural events involved in these might be important for motor-sensory temporal recalibration. On the basis of our data, we cannot comment on this last possibility. Our data speak only to the efficacy, or lack thereof, of neural activity that is causally related to events preceding motion onset.

Another possibility we should note is that, given the nature of our task, it is unclear if it is the planned time of ballistic action completion that is important for motor-sensory temporal recalibration, or if it is the actual time of ballistic action completion. As all the ballistic actions in this study were pre-planned, they would have had a planned as well as an actual time of completion, and these two times would have corresponded very closely. To identify which of these is more important for motor-sensory temporal recalibration, future studies could make the ballistic reach duration unpredictable, by applying an external disruptive force during the reach, or by moving the target button after the ballistic action has commenced. In either case, for our data the interval from movement termination to the adapting feedback was the most reliable predictor of whether temporal recalibration would occur, as opposed to the interval from motion instigation until adapting feedback.

In computational terms, we do not believe temporal recalibration necessitates that neural analyses must be sped or slowed in order to bring the timing of two neural events closer together in the brain. Instead, we believe this kind of effect can be driven by tightening or relaxing criteria used to judge temporal order or simultaneity (see Yarrow et al., 2011). Because this would not involve a shift in when signals are processed, this scheme could explain why temporal recalibration magnitude tends only to be a small proportion of the adapted offset (here adapting to a 200 ms lag resulted in a 60 ms recalibration, see also Fujisaki et al., 2004; Stetson et al., 2006). The implication is that while signal processing times are important for time perception (Roufs, 1963; Von Békésy, 1963; Wilson and Anstis, 1969; Bergenheim et al., 1996; Arnold and Wilcock, 2007), these are not the exclusive determinant. Rather, time perception can be modulated via an experience-based appraisal of timing relationships that can tighten or relax criteria about the point at which signals coincide in the brain (see Yarrow et al., 2011).

## Conclusions

Our data suggest that the critical neural relationship, in terms of timing, for determining the magnitude of motor-sensory temporal recalibration involves the sensation of having acted and the sensory activity triggered by that action. Our data therefore show that the times at which a person decides to act, and the timing of motor planning activity triggered by that decision, are inconsequential for motor-sensory temporal recalibration.

### Conflict of interest statement

The authors declare that the research was conducted in the absence of any commercial or financial relationships that could be construed as a potential conflict of interest.
